# What influences stroke survivors with physical disabilities to be physically active? A qualitative study informed by the Theoretical Domains Framework

**DOI:** 10.1371/journal.pone.0292442

**Published:** 2024-03-28

**Authors:** L. K. Kwah, K. Doshi, D. A. De Silva, W. M. Ng, S. Thilarajah

**Affiliations:** 1 Health and Social Sciences Cluster, Singapore Institute of Technology, Singapore, Singapore; 2 Department of Psychology, National University of Singapore, Singapore, Singapore; 3 National Neuroscience Institute, Singapore General Hospital Campus, Singapore, Singapore; 4 Department of Nursing, National Neuroscience Institute, Singapore, Singapore; 5 Physiotherapy Department, Singapore General Hospital, Singapore, Singapore; Swiss Paraplegic Research, SWITZERLAND

## Abstract

**Background:**

Although the benefits of regular physical activity (PA) after stroke are well established, many stroke survivors do not achieve recommended PA levels. To date, studies exploring determinants to PA have not used a behaviour change theory and focused on stroke survivors with physical disabilities. As a precursor to an intervention development study, we aimed to use the Theoretical Domains Framework (TDF) to identify factors influencing PA in stroke survivors with physical disabilities in Singapore.

**Methods:**

Between November 2021 and January 2022, we conducted interviews with 19 community-dwelling stroke survivors with a weak arm and/or leg. An interview guide based on the TDF was developed. We analysed the data deductively by coding interview transcripts into the theoretical domains of the TDF, and then inductively by generating themes and belief statements. To identify relevant TDF domains, we prioritised the domains based on the frequencies of the belief statements, presence of conflicting belief statements and evidence of strong belief statements.

**Results:**

Eight of the 14 TDF domains were relevant, and included environmental context and resources, knowledge, social influences, emotion, reinforcement, behavioural regulation, skills and beliefs about capabilities. The lack of access, suitable equipment and skilled help often limited PA participation at public fitness spaces such as parks, gyms and swimming pools (environmental context and resources). While a few stroke survivors expressed that they had the skills to engage in regular PA, most expressed not knowing how much and how hard to work, which exercises to do, which equipment to use and how to adapt exercises and equipment (knowledge and skills). This often left them feeling afraid to try new activities or venture out to new places for fear of the unknown or adverse events (e.g., falls) (emotion). For some, doing the activities in a group encourage them to get out and engage in PA (social influences).

**Conclusions:**

In stroke survivors with physical disabilities, environmental context and resources had a significant influence on PA participation, and this often had a spill over effect into other domains. Our results inform a complex behaviour change intervention to improve PA after stroke, and has implications for intervention design for people with physical disabilities.

## Introduction

The benefits of regular physical activity (PA) after stroke are well known. Regular PA reduces the risk of another stroke and improves the physical, mental and overall well-being of people after stroke [1, 2]. Despite recommendations of PA levels for people with disabilities or chronic illnesses (e.g., 150–300 minutes per week of moderate intensity PA [3], 6500–8500 steps/day [4]), most stroke survivors do not meet these PA guidelines [5]. Worldwide, stroke survivors achieved an average of 5535 steps in the subacute phase and 4078 steps in the chronic phase [5]. These numbers are similar in Singapore, with stroke survivors clocking a median of 4870 steps in the subacute phase [6], highlighting the prevalent issue of physical inactivity after stroke.

Factors influencing PA after stroke such as physical ability, environmental issues (access, transport, cost), social networks and participation in meaningful activities other than “exercise” have been reported in the United States, United Kingdom, Canada and Australia [7, 8], though stroke survivors in Singapore might highlight different determinants influencing PA levels, considering the differences in culture, clinical practices and health care systems. For example, a barrier such as cost of gym membership (reported by stroke survivors in the United States [9]) might not be as frequently encountered by stroke survivors in Singapore as all Singapore citizens and permanent residents are accorded $100 credit to access public gyms and swimming pools for free [10]. Considering the unique aspects of the public health infrastructure in Singapore, it is possible that barriers to PA faced by stroke survivors in Singapore might differ from others in developed countries [7, 8]. A clear picture of the barriers and facilitators influencing PA after stroke in Singapore is needed to design interventions that have a high chance of working and can be adopted into practice successfully [11].

To design an intervention that has a high chance of working and adoption into practice, it is necessary to have a clear picture of the determinants influencing the targeted behaviour [11]. While studies exploring barriers and facilitators to PA after stroke have been published [7, 8, 12], few studies have used behaviour change theories or frameworks to inform their results [12, 13] and focused on stroke survivors with physical disabilities. This group of stroke survivors are likely to be the least active, considering that stroke survivors consistently walked less steps per day compared to other chronic conditions (e.g., Chronic Obstructive Pulmonary Diseases, diabetes) and adults with physical disabilities/mobility limitations were the least physically active compared to adults with hearing, visual or cognitive impairments and adults with no disabilities [14].

In recent years, there has been an increasing demand to develop complex interventions to improve health [15, 16]. As health challenges become more complex, it is evident that health interventions targeted at changing human behaviour need to be multi-component, that is, involve a multitude of actions delivered by multiple individuals at multiple levels (e.g., policy makers at health systems level, health professionals at hospital level, patients at individual level) [16]. Although no single approach has been shown to be more effective than others, a framework/systematic approach for intervention development is often recommended to reduce research waste and increase the chances of producing a successful intervention [15]. In line with recommendations from the UK Medical Research Council to draw on theory when designing interventions [17, 18], we based our interview questions on the Theoretical Domains Framework (TDF) in order to map our findings back to the Behaviour Change Wheel (BCW) to consider the full range of intervention functions and select the most appropriate ones [11, 19]. Unlike other theoretical models of behaviour (e.g., the Theory of Planned Behaviour and the Health Belief Model) which might only explain some influences on behaviour [11], the TDF covers cognitive, affective, social and environmental influences on behaviour through synthesising 33 theories of behaviour and behaviour change categorised into 14 domains [19]. The BCW was also chosen due to its consideration of nine intervention functions and seven policy categories as elements to consider in the development of a complex intervention [11]. Using the TDF and BCW will therefore ensure a comprehensive and systematic process in identifying intervention functions that might be the most promising in encouraging uptake of PA by stroke survivors in Singapore. The process of identifying barriers and facilitators to PA *prior* to designing interventions will promote the design of interventions that are tailored to the specific context in which the behaviour occurs, and thereby increase the likelihood of behaviour change.

In this study, we aimed to use the TDF to explore barriers and facilitators influencing PA in stroke survivors with physical disabilities in Singapore.

## Methods

### Study design

We hereby report our study methods in accordance with the Consolidated Criteria for Reporting Qualitative Research (COREQ) checklist [20]. We conducted a semi-structured interview study using the TDF as a guide to develop the interview questions [19]. The behaviour of interest was in staying or being more physically active after stroke, in line with PA recommendations for people with disabilities or chronic illnesses [3]. This study is part of a larger body of research work aimed at developing a complex intervention to improve PA after stroke in Singapore. The study has been granted ethical approval by the Singapore Institute of Technology Institutional Review Board (project number: 2021104).

### Participant selection

Participants learnt about the study via a recruitment advertisement distributed via web-based methods (e.g., mailing list of stroke support organisations, social media, Whatsapp group chats), word-of-mouth and/or snowball sampling. We included participants who were aged 21 years or over, English-speaking, living in the community, had an affected arm and/or leg, and were interested in being more active. In accordance with the TDF guide, we estimated a minimum sample size of 10 participants but continued to recruit more in groups of three till data saturation (i.e., no new information on barriers and facilitators to PA) had been achieved [19]. Participants received a $20 voucher to reimburse them for their time and effort upon completing the interviews.

### Setting

Interviews with stroke survivors were conducted face-to-face in their homes, or virtually (via Zoom) depending on the preferences of stroke survivors in light of COVID-19 restrictions. In some instances, family members and/or caregivers were around during the interviews but they did not participate in the interviews, and their presence did not influence the sharing of the stroke survivors. Interviews commenced in November 2021 and were completed in January 2022.

### Data collection

Prior to the interview, stroke survivors completed a short face-to-face survey where baseline characteristics were obtained. Data included age, gender, ethnicity, employment status, years post-stroke, type of mobility aid, level of disability and perceived minutes of moderate intensity activity engaged in the past week. The level of disability was measured using the Modified Rankin Scale (mRS) that described the amount of assistance needed by stroke survivors to conduct their daily activities. To ensure standardised scoring of the mRS, we used the same structured interview that had previously shown increased agreement between raters [21, 22]. Moderate intensity activity was described as movements, or exercises that raise one’s breathing rate, or make one huff and puff [23]. The timeframe of one week was used as per recommendations in the World Health Organization [3] The interview guide (including questions and prompts) (see S1 File) was developed by the research team with reference to the TDF and BCW publications [11, 19, 24]. We used audio recordings to collect the data during the interviews. Field notes were documented after some of the interviews. Audio recordings were subsequently transcribed by an external transcription service.

### Research team and reflexivity

Interviews were conducted by the first author (KLK) and a research assistant (AK). For some interviews, undergraduate physiotherapy students were present (only one at a time) as the project was offered as a Honours Thesis project. Both KLK and AK had more than 10 years of clinical experience in the area of stroke. KLK (female, age 35–45) is an academic teaching at the local University and has a PhD in the area of stroke, while AK (female, age 30–40) is a senior physiotherapist with first class Honours from a BSc (Physiotherapy) degree. None of the participants were known to AK prior, while a few of the participants were known to KLK due to her involvement as volunteer in the Singapore National Stroke Association (SNSA). We did not think this would cause any substantial bias to the interviews as AK conducted most of the interviews, and KLK was not involved in virtual volunteering activities during the COVID-19 period which spans the duration of the study. Both KLK and AK did not have first-hand experience with disability, only second-hand experience having cared for people with stroke and other neurological conditions. Prior to the interviews, a 2-hour training session was held between KLK, AK and ST. All had access to the interview materials including the interview guide, TDF and BCW publications. ST had experience with developing interview questions, conducting interviews and analysing interview data [25]. During the training session, the team discussed each of the interview question in-depth, the use of prompts, and the relationship of the question to the TDF domains. The first batch of three interviews were reviewed by KLK, AK and ST to ensure questions were asked in an open-ended way to gather relevant responses to each of the TDF domains.

### Analysis and findings

After transcriptions of interviews, analysis of transcripts took a further 6 months and followed a two-stage process, using both deductive and inductive methods [19]. First, KLK and ST used a deductive approach to map four interview transcripts to the 14 domains of the TDF. Both KLK and ST open coded four transcripts (20% of all transcripts) line by line and met frequently to develop and revise the coding framework. Where there was discrepancy (e.g., whether a quote belongs in a TDF domain), both researchers discussed to reach consensus. In some cases, the participant’s quote was assigned to more than one domain [19]. If consensus was not reached, a third researcher (KD) was consulted. Results of these discussions were documented in a codebook to improve reliability of coding. We repeated the process till the Cohen’s Kappa (κ) between coders reached substantial agreement (more than 60%) [19]. KLK then coded the remaining transcripts using the codebook as reference. Second, KLK and ST used an inductive approach to generate themes and belief statements (i.e., a statement that provides detail about the perceived role of the domain in influencing the behaviour [26]) of the interview data within each domain of the TDF. To identify relevant TDF domains (i.e., domains that are likely to improve PA levels if targeted in interventions), we used the recommended three criteria: a) high frequency of the belief statements in domains, b) presence of conflicting belief statements, and c) evidence of strong beliefs that may affect the behaviour [19]. We regarded frequency as the number of different participants (out of 19) who expressed the belief statement rather than the number of times it was expressed [27, 28]. For the domains that did not meet all three prioritisation criteria, we deemed them as not relevant. We further categorised all domains (i.e., relevant and not relevant domains) according to level of importance: high importance (if all three criteria were met), moderate importance (if two out of three criteria were met), and low importance (if one or none of the three criteria were met) [27]. Relevant domains were equivalent to domains of high importance, while domains that were not relevant were equivalent to domains of moderate or low importance [27]. Microsoft Excel and Quirkos software were used to organise and analyse the data.

## Results

### Participants

Twenty-four participants responded to the recruitment advertisement, though only 19 completed the interviews. Reasons for non-participation included declining to sign the consent form (n = 2), no weakness in arm/leg (n = 2), and not being able to communicate in English (n = 1). The characteristics of our participants are shown in Table 1. Of the 19 participants interviewed, most were male (53%), of Chinese ethnicity (89%) with a mean age of 55 years (range 42 to 68 years). All were in the sub-acute to chronic phase post-stroke (median 9 years; IQR 4 to 13 years post-stroke). More than half of our participants (58%) had slight disability (score of 2 on Modified Rankin Scale), and more than half (74%) frequently used a mobility aid. Only a small number of participants (16%) reported having met 150 minutes of moderate intensity PA per week. Interviews lasted typically for 30 to 60 minutes. Findings of the transcripts were summarised and returned to participants for feedback in a separate co-design workshop. We recruited till data saturation was reached, and determined this by reviewing transcripts concurrently in batches of three [29]. In the last three interviews, all relevant quotes were coded to theoretical domains of TDF that already had quotes from prior interviews. As no new information was gained, we stopped at 19 participants.

**Table 1:** Characteristics of participants with stroke.

Characteristics	All participants (n = 19)
Age (years), mean (SD and range)	55 (SD 7.03; range 42 to 68)
Gender (male), n (%)	10 (53%)
Ethnicity, n (%)	
Chinese	17 (89%)
Indian	2 (11%)
Employment status, n (%)	
Retired	6 (32%)
Student	1 (5%)
Employed	9 (47%)
Unemployed	3 (16%)
Time post-stroke (years), median (IQR and range)	9 (IQR 4 to 13; range 1 to 37)
Modified Rankin Scale, mean (SD and range)	3 (SD 1; range 2 to 5)
Modified Rankin Scale, n (%)	
1 = No significant disability	0 (0%)
2 = Slight disability	11 (58%)
3 = Moderate disability	3 (16%)
4 = Moderately severe disability	4 (21%)
5 = Severe disability	1 (5%)
6 = Death	0 (0%)
Most frequent mobility aid used, n (%)	
No aid	5 (26%)
Walking stick/Quad-stick	6 (32%)
Motorized scooter/Electric wheelchair	6 (32%)
Manual wheelchair	2 (10%)
Minutes of moderate intensity activity engaged per week, n (%)	
Less than 150 minutes	16 (84%)
Up to, or more than 150 minutes	3 (16%)

### Inter-rater reliability

Inter-rater reliability between two raters (KLK and ST) was assessed from four interviews, and reached agreement of 76% (95% CI 69 to 83%) (considered “substantial agreement” [30]) after four rounds of coding and discussion.

### Relevant TDF domains

**Fig 1:**
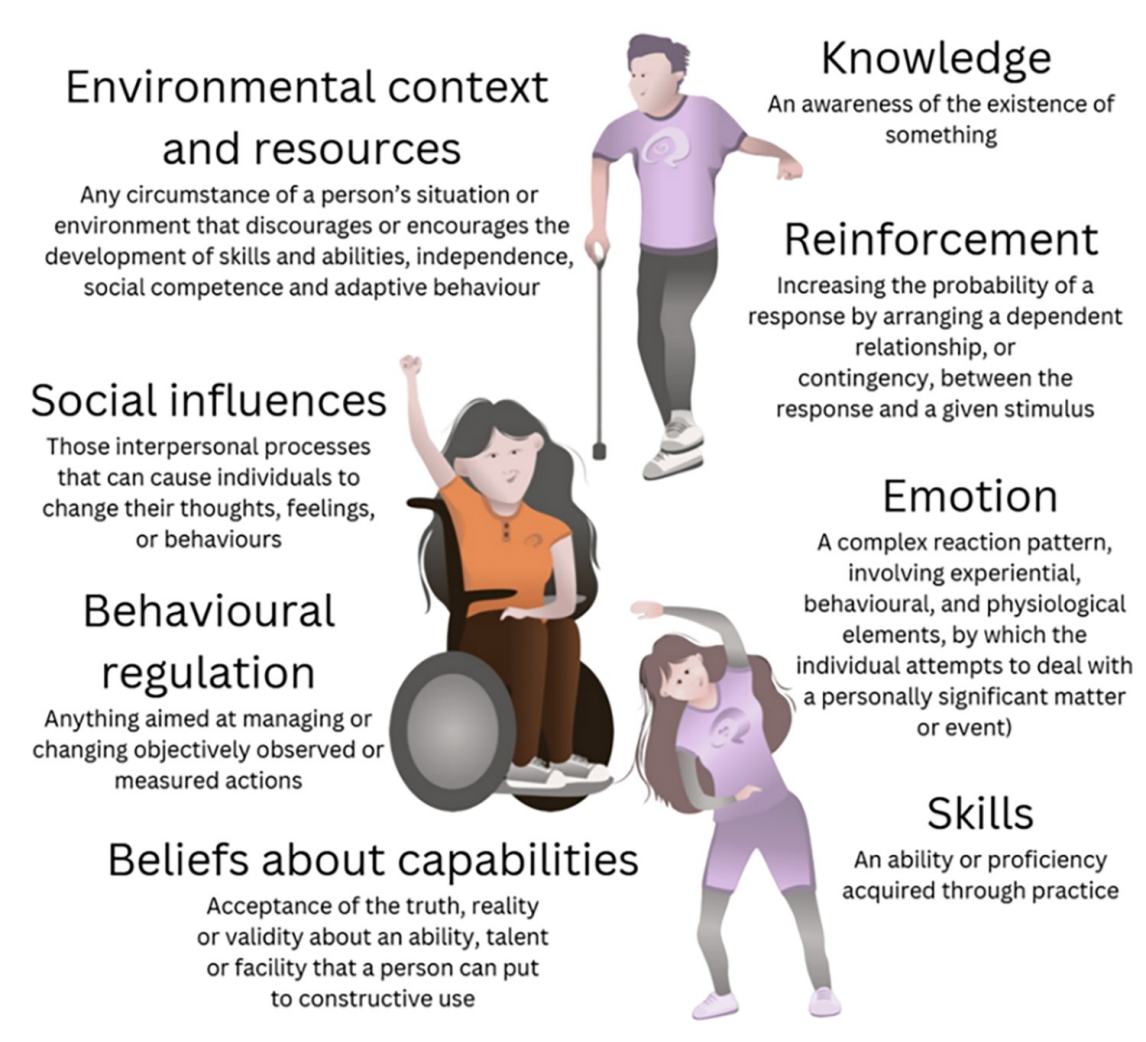


In total, 497 quotes from 19 interviews generated 54 belief statements, coded into 14 domains of the TDF. Only 355 quotes were regarded in the frequency count as we ensured the number reflected the number of different participants who expressed the belief statement rather than the number of times it was expressed. Eight relevant TDF domains were identified (Fig 1). Relevant domains were likely to influence behaviour and considered of high importance (Table 2). These domains included environmental context and resources, knowledge, social influences, emotion, reinforcement, behavioural regulation, skills and beliefs about capabilities (Table 3).

**Table 2:** Level of importance of each TDF domain based on frequency and strength of belief statements, and conflict in belief statements.

TDF domain	Frequency of belief statements	Conflict in belief statements	Strength of belief statements	Level of importance^a^
Environmental context and resources	63	Presence	Strong	High
Knowledge	44	Presence	Strong	High
Social influences	33	Presence	Strong	High
Emotion	34	Presence	Strong	High
Reinforcement	32	Presence	Strong	High
Behavioural regulation	30	Presence	Strong	High
Skills	19	Presence	Strong	High
Beliefs about capabilities	18	Presence	Strong	High
Beliefs about consequences	27	Absence	Strong	Moderate
Goals	15	Presence	Weak	Low
Intentions	10	Presence	Strong	Moderate
Optimism	10	Presence	Weak	Low
Social/Professional role and identity	16	Absence	Weak	Low
Memory, attention and decision processes	4	Absence	Weak	Low

^a^The TDF domain was considered of high importance if all three criteria were met, moderate importance if two out of three criteria were met, and low importance if one or none of the three criteria were met.

**Table 3:** Relevant belief statements and sample quotes tagged to the TDF domains.

TDF domains	Belief statements	Sample quotes (Participant number)	Frequency (out of 19)
Environmental context and resources	When I wanted to be more physically active, I could not due to issues related with COVID-19.	“…because of Covid, we cannot go [out] most of the things, you know, those organised outings… we cannot meet other people or friends or outings.” (P2) “The only thing that change is I don’t go to gym and also library, I don’t really go now. Because one thing is the COVID cases, at one period of time, it went up quite high.” (P11) “Before the pandemic, actually I go every week. The ActiveSG one… but because of the pandemic strike, I stop going. I wanted to, but because of the bookings. If I want to go to the gym, I need to [book], so very difficult. I remember last time I can walk in […] very troublesome.” (P12)	12
	When I wanted to be more physically active, I could not due to issues with use of equipment.	“…it’s quite difficult for person with disability to join [outdoor fitness zones] [. . .] I can’t find even one [outdoor fitness equipment] I can sit down and start doing. … The wheel… I can’t sit on the seat because it’s not very accessible to me. Because I have to get down. Then I have to shift myself in right? So when I grab hold of the wheel, there’s nothing to tie the leg… Then those who are doing the hand…sometimes too tight, and I can’t put the hand on the wheel.” (P4) “The handwheel cannot. Then there is the leg press I cannot where you use your weight. That one not too bad, still ok, but one thing is not able to hold my feet on the pedals. Which also stopped me from doing it. So if they have something that can hold my feet, I will go… The equipment there are suitable for normal person, but it’s not suitable for weak people. For stroke survivor. So I tried you know..there is the one where you step on top then you go up and down—I tried that, but I cannot. In the end, the word safety still comes to me. The word safety is still haunting me. Yeah, so I also worry, so also cannot. So actually [outdoor fitness equipment] are more for normal person.” (P12)	12
	When I wanted to be more physically active, I could not due to issues with access and use of physical environment (in terms of space).	“The place must be handicap friendly and the area that is for handicap they need to really make sure that it’s for handicap only […] We can’t use it [accessible toilets] at all because it’s being occupied by them [able-bodied people].” (P5) “[…] Normal swimming pool stroke people not quite suitable, because even it’s just a narrow pool you can’t go down to the pool because the steps beside are not suitable to go down.” (P6)	10
	When I wanted to be more physically active, I could not due to lack of physical assistance.	“I think hydrotherapy like swimming […] is actually very helpful but in my case, I definitely need help getting out of the pool. . .So these are certain things that are holding me back from doing.” (P3) “But if let’s say I were to try something new. I have not done it before. I also don’t know how to go about it. It’s like…, we have no knowledge about stroke, it’s just that unfortunately we got stroke. So … just as any other normal people that have no knowledge about what stroke is, how to go about to tackle the issue of stroke or to improve ourselves. Totally ignorant about what is going on. So unless someone taught us or unless like sometimes I happen to go to YouTube and watch some YouTube from overseas that teach certain exercise then if I feel that I’m confident enough to pick it out and do it, I’ll do it. But if I don’t feel confident enough that their exercise, not easy to do it, I won’t force myself to do it. I go and search, I just kind of play around with YouTube.And then to see what are the physiotherapy around the world. But sometimes all these videos, because it’s videos… you don’t really have a live person that taught you. It’s like you see already, you can kind of know and don’t know because it’s like no hands on. Then therefore it’s a bit hard to know how to do it also.” (P9) “No is more of no professional around. So should anything happen, you just [stuck] you know. […] If you are not familiar, no confidence and you dare not. The more you get scared then it [adverse event] really happen. […] Someone has to be there to encourage us [. . .] (P18)	11
	When I wanted to be more physically active, I could as there were minimal issues with access and use of physical environment (in terms of space).	“[…] the swimming pool, because they got a ramp so it’s easier for people like us. But not everybody can have the strength or power to get out of the pool using the steps […] (P7)	6
	When I wanted to be more physically active, I could as there were minimal issues with use of equipment.	“[…] the equipment is quite—I mean, user friendly la. And moreover, I think the gym is also quite user-friendly. Well, okay, the thing is for me, sometimes I will take a cab to go there, but sometimes I will take a bus. That means bus, once I alight right, I just need to walk about 10 to 15 minutes to reach the [name of gym] itself. And I think it’s quite wheelchair-friendly. As mentioned, because—I mean the objective of the person who is using it. For me, I want to strengthen myself, so whatever equipment that I have right, even when I go to the normal one, leg press right, instead of using two legs, I will try to use one leg to press now. Because mainly I want to strengthen my left leg more, so I will be using one leg. So if I’m back to the disabled one right, then I’ll be using one leg, I’ll try to be using one leg. . . because one thing is that they actually have instructions, if I don’t remember wrongly, they actually have instructions on the equipment itself, telling people how to use and what is it for [. . .]’ (P11)	3
	When I wanted to be more physically active, I could not due to lack of money.	“I really need company. Then, how to say? Also need money. You need to travel, yeah.” (P8)	3
	When I wanted to be more physically active, I could not due to other competing priorities/commitments.	“Okay, so a group of them [stroke survivors], so they still swim […] They even go to the gym. The thing is that I didn’t join them because of my own commitment because we work on weekdays.” (P7)	3
	When I wanted to be more physically active, I could not due to weather.	“Being active. . .Yes. I try unless it’s raining heavily. If it’s raining heavily, then I don’t go […].” (P1)	3
**Knowledge**	I am aware of the consequences of being physically inactive.	“Yeah, because our mind is not working, the first thing. Then we will like bound to be quite moody […].” (P2) “You got to be active. If not, it will not come back. Mental more important, more on mental. Once you’re active, when you see the improvement then you are more motivated to go on. As you see, if you are not active. If you don’t use your muscles, your muscles will shrink. The muscle will shrink and then your brain will get hardened. You don’t go for your exercise slowly would be stiffen up. You want to move also you cannot move.” (P5) “I’ll become bedridden every day. And then sooner or later, it will affect my ability to think, because then I don’t think a lot. I don’t use my brain then it affects my whole being, my morale.” (P9)	18
	I am aware of the benefits of being physically active.	“Okay, also to keep us kind of like alert and alive… also if I don’t walk more, it affects my bowel, I won’t be able to go toilet. Yeah, since stroke, I have very bad constipation. So I was told it’s because don’t walk enough. …physiotherapy or walking, …the more you do it, the better you get at it.” (P9) “Okay first, exercise. If you enough exercise, you can cut down your cholesterol. You can cut down your high pressure. You can cut down your body sugar level.” (P10) “Actually for me being active will help us in terms of our health itself. I would say, like mental health and also physical health. When we keep active, I mean this is what I learned from what I Google la, is that when we keep active, our brain will be more active. And not like going to be dying…. And then physically when we keep activet, it actually help me in my strengthening. And also because stroke survivor, if I’m going to add stretches or exercise, my whole body will be very tight and stiff already. So I will need to do all this, to ensure that I don’t accumulate all this sort of tightness itself, if not I’ll be in pain. . .Health-wise is that also let’s say I keep myself active, I can prevent other illness from coming in. Because […]if I keep active, for example for my heart, it can help make my heart healthy, instead of being with other illness. . .So when I keep active, it can help me toensure that my mind […] is healthy right, in a way.” (P11)	11
	I am not aware of any physical activity (PA) guidelines.	“How long? I’m not, I don’t really have analysed that, so. I don’t really know how long. I will say maybe at least half an hour to an hour minimum. In a day, yeah. How hard? No, I don’t go by that kind of gauge. I usually set my own target and go, so it’s like if I have to do like 10 set of the exercise per day. Then I finish all my 10 set. Yeah, then is like I finished up my 10 set, I finished up. Then if I can do more that day then I’ll do another 10 set maybe in the afternoon or in the evening.” (P9) “I do not have this amount, […]I like to I keep it more like everyday, sometimes maybe a little bit better, sometimes maybe a little bit lesser like that, but I don’t have steps to count on. No, I don’t do that.” (P20)	**7**
	I am aware of some physical activity (PA) guidelines.	“I think to be effective, minimum is to be 30 minutes and above. At least three times a week for 30 minutes, unless you’re doing above one hour, maybe then we have to do two days or one day per week. I think so far that after stroke, how far I would think that I have walked with a group of people for more than 30 minutes non-stop walking, I think that should be good enough. The length of the exercise.” (P6) “At least if we take the MOH guideline right, 10,000 steps. Ok, at least 10,000 steps per day. At least 10,000 steps per day. Tracker, 10,000 steps tracker. . .You don’t want to have vigorous exercise, at least you go for a ten thousand walk, which is a minimum requirement by MOH, which will help you to sweat it out.” (P12)	**7**
	I do not know of various PA opportunities for stroke, or how to access them.	“I have been asking around how do I sign up for swimming lesson but nobody seems to know about it. They say that now with Covid everything is stopped. Because I used to be a swimmer and I love to swim so I’m hoping to see whether I can use back some function. So before the stroke, [I was] swimming quite a bit. And then ever since after the stroke, [I] haven’t really swum. Cannot swim at all because my left side is very weak.” (P16)	1
**Social influences**	Friends/colleagues have a positive influence on me staying/becoming more physically active.	“When you are in a group, then you see each other then you encourage each other, motivate each other. Then you see people cannot do then you encourage them or you cannot do then you see people can do then you start to wonder why people can do and you cannot do.” (P4) “Of course in the group, besides exercise physically, you actually have a conversation. You actually in a ommunication with other group, so that helps in so called that interaction with others. There’s also kind of social interactive. That’s why it’s help not only physical, buy also mentally. We start to encourage one another. This is also more important, quite important.” (P6)	15
	My family have a positive influence on me staying/becoming more physically active.	“No, they always help me, in case I am a little lethargic then my husband will keep pushing me.” (P1)	6
	My family have a negative/no influence on me staying/becoming more physically active.	“My [family member] will stop me from [being] more active. Yeah because whenever I want to go down to … our new mall here has a [name of shop]. So I wanted to go there and see. She just say because of the pandemic, why, she will ask me why I want to go out because wherever I want to go is a bit troublesome because need to wear the bra. Yes. Then my shoes, I need to wear my ankle support to go down [. . .] Stops me sometimes.” (P8)	4
	No one influences me to stay/become more active.	“Uh, they don’t encourage me, but they do not discourage me. Yeah, neutral. So there’s no one who encouraged me or discouraged me, and actually maybe for my personality right, even if people discourage me, but if I want to do it, I will still continue to do it. Because for me, actually I think it’s important for me la. . . I mean to me is just say I think this is important to me right, I mean even other people discourage me, I don’t really agree right, but I think it’s better for me, I will still be doing it. Yeah.” (P11)	3
	Strangers have a positive influence on me staying/becoming more physically active.	“I don’t think I’m conscious or anything because I know I have walk and I have to wear an AFO. And people also have [been] quite encouraging that way. In my condominium, most of them they keep motivating me […] I do all my bathing activities. Then I go down again in my condo. I go down, meet everybody, meet little kids, but I’m very fond of kids, small kids. I have lots of small friends, young friends who go meet them. Say hi to everybody, then go to gym. We have a nice gym in our condo, outside gym” (P1)	2
	Friends/colleagues have a negative/no influence on me staying/becoming more physically active.	“Friends…we hardly meet each other nowadays. Everyone got their own things to do. Then usually they stay at home. Just WhatsApp message or what? Just talk only… I don’t depend on friends.” (P2)	2
	Strangers have a negative influence on me staying/becoming more physically active.	“Some of them still look at you and say " May I help? May I help?" Of course they meant good, right? But there are people who think that they are no longer in that league, Because discard, that kind of thing. I do feel it. I do feel it. I do feel it that part. The way they look at you and the way they talk. Not looking down but you can sense it. I try not to focus on that. Honestly, I try not to focus. I felt that this would drive me down. And I think that it’s because they are ignorant and they do not know. That’s why they have this kind reactions.” (P18)	1
**Emotion**	I feel a sense of accomplishment/satisfaction/success when I think about being physically active.	“Yep, and you are more independent in that sense because eventually I got back my driving license after. Six after six months or so. . .go out meet friends for coffee again. Rather than depending on someone to send you to rehab for therapy. Well, yeah, I think that really helped a lot.” (P3) “To me is like try to get back your life. Like you are still capable of doing things. By doing things and also adapting. Using ways to adapt, you find well, you can do it. then satisfaction.” (P18)	12
	I feel pleasure when I think about being physically active.	“Yes, I walk. That gives me a lot of pleasure. . .Yes, I like enjoying the nature.” (P1) “Of course, it makes me happy. Makes you cheerful. Mentally, physically, I’m very happy It helps, it helps me. Just imagine lying on the sofa, watching TV, you think it helps anot? […]That’s why when a person is motivated, you will be able to do faster. Yeah. It makes you relax.” (P12)	10
	I feel afraid/scared when I think about being physically active	“Yeah, I mean it’s as a group exercise or whatever, I think I’m quite confident because we are quite well protected in the sense. Alone, if the environment is new to me, I’m still a lot of doubt.” (P6) “But if she’s not around then I alone I dare not venture out. . .To do all these. Because no matter how sometimes you know, our stability is not there sometimes walk, walk, walk, suddenly the leg feels weak and then you can fall down. So I really didn’t want to. I don’t want to have any accident, at this point, no one wants to go to the hospital, it’s just not convenient.” (P9)	9
	I feel discomfort/ helplessness/ frustration when I think about complications limiting me from being physically active.	“No, it’s not very hard, but if my inversion problem was not there then probably I could have tried even swimming or Aqua therapy and all, but I can’t go down without my AFO like if I have to get into water, then I have to remove the AFO. I’m scared that I’ll slip and fall.” (P1) “I can’t walk already. Or my legs give me some pain because I’m wearing my AFO, you see. Problem. I stop. So I’m now being bound by this.” (P18)	3
**Reinforcement**	To be more physically active, opportunities to improve self physically, mentally or emotionally keep me going.	“I want to be active because I want to keep my mind thinking. . .Being active like we can talk to our friends. And organise something to achieve. Like I like to organise outing or whatever in the committee, in my association committee. . . .so we can keep our mind thinking.” (P2) “I think it’s more intrinsic. If I’m active I’m able bodied, I can walk better, I think that’s the motivation for me. Cannot walk then I’m stuck in the house, that kind of thing, that’s very bad. That is my motivating point, to push myself such that I will walk for tomorrow.” (P15)	11
	To be more physically active, social opportunities/ opportunities to connect keep me going.	“I want to be active because I want to keep my mind thinking. . .Being active like we can talk to our friends. And organise something to achieve. Like I like to organise outing or whatever in the committee, in my association committee. . . .so we can keep our mind thinking.” (P2) “Yeah. Volunteer, what type of volunteer you doing? I can say, a lot of volunteer, you can do it. You want or don’t want only. Because of the volunteer, first, you go and make a lot of friends. You can see a lot of things, different things. That one also good for us. Not say the stroke only. Others also same, need to do more every day, occupy yourself […].” (P10)	7
	To be more physically active, vouchers/financial incentives do not keep me going.	‘But then it [voucher/financial incentive] doesn’t appeal to me. I don’t know why, but it appealed to my sister there. I don’t know because like her age 60 over years old maybe she don’t work so she got more time doing all these collect points and walk.” (P4) “I believe they help, but I do not know because I’m not the active senior, so maybe we don’t need that, but I even have problem to put on a watch myself and put on all these things. Okay, I think that if I can do it myself, I mean to me it’s not important that how much I walk or record or to show. I mean I have one group chat from [insert organisation]and the people there they are very active, of course it’s good for them. They share how much they walk a day, how much energy whatever they use all these things, but they have the equipment. I mean to have the equipment is not a problem, but I used to have one watch before but I didn’t wear it, I didn’t use it. To me if I walk that’s good for me, that enough.” (P6)	6
	To be more physically active, consequences keep me going.	“I wouldn’t say that it’s a habit but sometimes I need to remind myself or force myself to get out, you know… to work out a sweat. One instance is when your pants are getting tight.” (P3) “… I still force myself to do a lot exercises because I feel that if I don’t do it my leg will worsen. So I have to do more in order to improve myself…I know that if I don’t want to keep myself active, I will end up very demoralised […] I will end up just lying down on the bed, as good as the dead person like, you know, [being] bedridden then I can’t do anything and because I have seen other people that didn’t want to do anything, they give up and then before you know it, … their body, their brain, their life gone. So I don’t want that to happen to me.” (P9)	4
	To be more physically active, vouchers/financial incentives keep me going.	“Oh yeah I mean, what about I’m out walking anyways? So I’ll try to score some vouchers […] (P3) “I think the government should do more because you have the tracker everything right? But there is nothing for the [people with] disability, right? Because you give people, for example a normal person like maybe 1000 steps. Then you are given a voucher. Maybe for a person with disability, we cannot take 1000 steps. Maybe only can take 5000. Compared to the normal person. Or rather people who like me can’t take steps right? Maybe you can other form of encouraging or maybe the number of hours work out or whatever.” (P4)	4
**Behavioural regulation**	I keep track of how much I am doing in terms of staying/ becoming more physically active	“I have Google Fit app on my phone, on Steps app which counts my number of steps and how much I have walked.” (P1) “At least half an hour, you cannot do much. If you do much yes, after half an hour, stop another 15 minutes to 20 minutes. That’s the sign I give.” (P10) “I try to slowly slowly do 10 times, then rest a while. 10 times, then rest a while until 60 times or 80 times.” (P17)	14
	I stop when I feel tired/pain, or don’t feel well.	"I just go with the day’s flow so if I that day don’t feel too good or feel very tired. I don’t force myself to do it. . .” (P9) “By feel and energy level. How much can I give, or do? If I’m low level, I cannot do so much and then I’ll not able to find my way back. I must leave some energy to go home. I cannot just do it all and then I achieve 10,000, but I cannot go back. So you must have balance somehow.” (P15)	10
	I do not keep track of how much I am doing in terms of staying/becoming more physically active	“Yes. To me I have this mentality that is you don’t care how I do, as long as I do, I get it done, I’m done. So I don’t really so particular of the timeframe.” (P7) “Most of the time I don’t use. No, I don’t have. I don’t have. For example maybe sometimes let’s say I walk around the estate, sometimes there will be some people there, […] say Mr you want to stop, take a rest first, I say no I still can walk, usually that’s my way, walking.” (P20)	3
	I do not keep track of how hard I am working in terms of staying/becoming more physically active	“Now I don’t push myself. Now is leisure swimming already. Because I don’t go for competition anymore.” (P5)	2
	I keep track of how hard I am working in terms of staying/becoming more physically active	“Yeah, I use my pulse. This is how I feel. I’m trained to do all this. With my own manual heartrate, checking pulse. Yeah you can test. Ok so you just put here. . . This is the resting, quite low you know, I can go as low as 60 to 50, I’m quite surprised. If I feel tired and lethargic, there are two things, I also have this blood pressure machine. So every day I will assess, morning, noon, before training after training, test my heart rate, my blood pressure. So during my blood pressure I can get my heartrate as well.” (P13)	1
**Skills**	I do not have the skills to stay/become more physically active.	“I have not come to the extent that I really feel the exercise is hard for me because when I do all these exercise, normally I have a qualified physiotherapist or whoever around, and normally they are always very protective, a little bit they said oh, you should be tired, you should stop. Even I know that I can go further, but normally to safeguard they say okay stop now, I think you’re tired.” (P6)	7
	I do not have the skills to select and use equipment.	“[…] Like gyms wise, you can’t do that much because one hand cannot lift. . .I already tried […] the thing is our so called disabled arm, we can’t do much. People like us, even now you put on those like strap to the hand bicycle, no use, we’ll trip. . . .That’s why they should have something that have more able to help people to do it. Maybe something that will be smaller length. So then we can just grab and hold so that you somehow will lift up right, so it’s still within the safety net. When I do it, when I lift it up, it will just follow up, just like our rock climbing so it will go up, so if anything happen like drop load, it still lock it, so have a safety net, so this thing will not land on my leg. . .Like myself my right side cannot use. If you have the strap to it, so when I lift up, it won’t be so hard because we were starting to the speed or the control that so help me to simulate equally with the weight we lift. . . So I think if you do something like this one, it will be more wonderful, just like the treadmill is the same thing. Something that can help us control the speed. We try to move on treadmill, but it might be a bit …not so tightened. You know the treadmill when you run, the more the treadmill is there the speed is there. But if you don’t want to move, if move too fast cannot. So I hope, it has to be much much slower or there is a limit so that even if we push stop, we won’t fall down. Because once you step out the treadmill once you cannot pace with the starting, you will trip.” (P7)	6
	I have the skills to be stay/become more physically active.	“But now we change, because I need to—I mean, strengthen most on my hip right, so I’ve been doing—I mean a suggestion, I mean I’ve bought a weight and been doing at home [as unable to go to gym].” (P11)	6
**Beliefs about capabilities**	I am not confident that I can engage in more PA (i.e., adapt exercises).	“Not that I can think of now because otherwise I will have done it. I have tried a few times with the stroke association. They also have virtual exercises, I have tried but just don’t motivate me. I have even tried using the YouTube, those sit down exercise. . .I don’t have specific, I just find exercises that I can do. I mean those who are more on sitting down, I don’t like those […]. I like the intensive one, then you just do short like 5, 10 minutes can already. (P4) “Yeah, vigorous exercise. I don’t think I can really ever do it, the only thing I can do is walk, walk and walk. There’s nothing much I can do.” (P12) “Well, as I said, I used to be very confident, I can do things, but when you can’t do and fall down, then you stop to try to do it. . . I really cannot answer because every time when I work towards being active, I came out with another health challenge. So I really cannot answer because just right now, until I can about to be able to walk, I am due for another surgery. Surgery after surgery so I don’t know what. Every surgery is a reset. Reset to back to square one. I can’t explain. I can’t even answer what do I want to do and what’s going to happen, because again, it depends on the result of the surgery. Yes, in fact there was what all my current OT, PT has been advising because they also don’t know what to teach me. It all depends on the result.” (P16)	9
	I am confident that I can engage in more PA (i.e., adapt exercises).	“I work, I do house work. I do exercise. . .I’m a security officer so I do patrolling. I monitor CCTV… Exercise can be anything.You sweep the floor, you mop the floor in your house that is exercise to me. So that is not an issue what, everyday you can do. Or you just have to come down, walk down, walk to the coffee shop, have your breakfast, or have your lunch or whatever it is, that is also exercise. As long as you move. You don’t keep lying on your bed, that is exercise already. You can have so many things to do.” (P5)	4
	I am accepting of my present capabilities.	“No, I think none of us would be happy with our activities level because before my stroke I’m a super active person and I’m a very independent person. So right now, I think I’m only doing half of what I used to do. In my understanding, it’s not possible, so I don’t force myself to put high expectations that I will get back to what I used to be. I try but I know that it’s not possible. I can never be like what I used to be.” (P9)	3
	I am not confident that I can engage in more PA (i.e., use equipment).	“I am not confident of using the treadmill. Prior to my stroke, I used to run. . .But now, I’m a little underconfident. . .Yes. I wish I could run again.” (P1)	2

#### Environmental context and resources

Many factors influenced stroke survivors’ participation in regular PA, of which environmental context and resources played a significant part. Most stroke survivors reported issues with access and use of public spaces, and these issues included lack of ramp access into swimming pools, wheelchair access into public spaces, inability to use accessible toilets (either due to toilets not working or being used by abled people) and the lack of accessible parking lots. Exercise equipment at the outdoor fitness zones and gyms were often deemed unsuitable, with most stroke survivors reporting difficulty in positioning their affected arm/leg onto the machines and getting onto the seats of the machines which are either too small or were not adjustable to allow use of the machines. In these instances, physical assistance (both skilled and unskilled) were deemed useful (e.g., having someone to assist them into the pool, or to guide them on the use of the machines). For the few who reported having minimal issues with access and use of space and use of equipment, they visited the swimming pool or the gyms in groups (sometimes supported by volunteers) and also learnt to rely on adaptive equipment (e.g., ankle-foot orthosis) or adapt everyday objects (e.g., using milk bottles as weights) to allow them to engage in more PA. Unsurprisingly, the COVID-19 pandemic made it even harder for stroke survivors to engage in PA due to the cancellation of group exercise classes and social outings. Although virtual activities replaced the physical activities, it was not possible to replicate fully the atmosphere and physical connections that one made with others in a face-to-face setting. There were also limited gym bookings available due to capped group sizes, and the risk of infection deterred some stroke survivors from heading out of their homes. Other PA barriers related to environmental context and resources also included the lack of finances, competing priorities/commitments and poor weather though these were much smaller obstacles compared to the access and use of public spaces, and use of equipment in public spaces.

*“[…] Normal swimming pool stroke people not quite suitable, because even it’s just a narrow pool you can’t go down to the pool because the steps beside are not suitable to go down.” (P6)*

*“…it’s quite difficult for person with disability to join [outdoor fitness zones] […] I can’t find even one [outdoor fitness equipment] I can sit down and start doing. … The wheel… I can’t sit on the seat because it’s not very accessible to me. Because I have to get down. Then I have to shift myself in right? So when I grab hold of the wheel, there’s nothing to tie the leg… Then those who are doing the hand…sometimes too tight, and I can’t put the hand on the wheel.” (P4)*

*“…because of Covid, we cannot go [out] most of the things, you know, those organised outings… we cannot meet other people or friends or outings.” (P2)*

#### Knowledge

While some stroke survivors had knowledge of the PA guidelines in terms of duration and number of steps to achieve for health benefits (e.g., 30 minutes or 10,000 steps daily), none mentioned the intensity of PA needing to be of moderate intensity. Thus there appeared to be a greater awareness of the recommended duration or quantity of PA (i.e., how much one should be working) than the intensity of PA (i.e., how hard one should be working). Almost all stroke survivors knew about the benefits of PA and consequences of physical inactivity. The knowledge domain was closely linked to the skills domain. Stroke survivors highlighted the lack of information on appropriate PA opportunities, and not knowing what exercises and machines are appropriate for them to use as factors influencing their engagement in PA.

*“How long? I’m not, I don’t really have analysed that, so. I don’t really know how long. I will say maybe at least half an hour to an hour minimum. In a day, yeah. How hard? No, I don’t go by that kind of gauge. I usually set my own target and go, so it’s like if I have to do like 10 set of the exercise per day. Then I finish all my 10 set. Yeah, then is like I finished up my 10 set, I finished up. Then if I can do more that day then I’ll do another 10 set maybe in the afternoon or in the evening.” (P9)*

*“I have been asking around how do I sign up for swimming lesson but nobody seems to know about it. They say that now with Covid everything is stopped. Because I used to be a swimmer and I love to swim so I’m hoping to see whether I can use back some function. So before the stroke, [I was] swimming quite a bit. And then ever since after the stroke, [I] haven’t really swum. Cannot swim at all because my left side is very weak.” (P16)*

#### Skills

As the quotes on exercises and machines were related to their ability to carry out a physical task that would improve with practice, most of the quotes were coded in the skills domain. Some of these skills were related to pushing into intensive exercise, progression and adaptation of exercises, problem-solving in new environments, and using machines in gyms and outdoor fitness zones. Without the knowledge and skills, stroke survivors shared that they were either still doing the same exercises/activities previously prescribed by their physiotherapists in the past, intentionally limiting activities in new environments (e.g., crossing the roads, visiting busy malls, getting on escalators), or not using any of the equipment such as treadmills, cycle machines and leg press in gyms and outdoor fitness zones.

*“I have not come to the extent that I really feel the exercise is hard for me because when I do all these exercise, normally I have a qualified physiotherapist PT or whoever around, and normally they are always very protective, a little bit they said oh, you should be tired, you should stop. Even I know that I can go further, but normally to safeguard they say okay stop now, I think you’re tired.” (P6)*

*“[…] Like gyms wise, you can’t do that much because one hand cannot lift…I already tried […] the thing is our so called disabled arm, we can’t do much. People like us, even now you put on those like strap to the hand bicycle, no use, we’ll trip… . You know the treadmill when you run, the more the tread is there the speed is there. But if you don’t want to move, if move too fast cannot. So I hope, but it has to be much much slower or there is a limit so that even if we push stop, we won’t fall down. Because once you step out the treadmill once you cannot pace with the starting, you will trip.” (P7)*

#### Social influences

Stroke survivors stated both positive and negative influences of people around them in influencing their PA, though there were more positive than negative influences. Most stroke survivors shared on the physical, emotional and mental help received from family, friends/peers, colleagues and strangers which have motivated them to engage in more PA, be it walking outdoors, visiting the gym, swimming, returning to work activities, and attending social events/outings organised by SNSA. Negative influences were few, and were associated with nagging, lack of time to assist and lack of motivation to exercise on the part of the family and friends. Here, it is interesting to note that although some stroke survivors enjoyed the company of their family and friends during PA, they also expressed a preference to be alone at times so that they can focus on exercising (as they tend to talk more than exercise more in the presence of company).

*“Of course in the group, besides exercise physically, you actually have a conversation. You actually in a ommunication with other group, so that helps in so called that interaction with others. There’s also kind of social interactive. That’s why it’s help not only physical, buy also mentally. We start to encourage one another. This is also more important, quite important.” (P6)*

*“My [family member] will stop me from [being] more active. Yeah because whenever I want to go down to … our new mall here has a [name of shop]. So I wanted to go there and see. She just say because of the pandemic, why, she will ask me why I want to go out because wherever I want to go is a bit troublesome because need to wear the bra. Yes. Then my shoes, I need to wear my ankle support to go down […] Stops me sometimes.” (P8)*

#### Emotions

Both positive and negative emotions were reported to influence PA. Stroke survivors reported gaining pleasure from socialising in a group, getting out and about in nature, and experiencing the endorphins from exercise. They also reported an increase in their confidence levels when they became more independent in their daily activities, or learnt to do a new activity in an adapted manner without requiring as much help as before. Where negative emotions were reported, these included the fear of falling or other adverse events, fear of the unknown and having doubts/worries on what to expect, particularly in new environments. Some stroke survivors also expressed discomfort/helplessness/frustration when they considered the complications limiting their PA (e.g., pain, spasticity and bladder issues).

*“To me is like try to get back your life. Like you are still capable of doing things. By doing things and also adapting. Using ways to adapt, you find well, you can do it. then satisfaction.” (P18)*

*“No, it’s not very hard, but if my inversion problem was not there then probably I could have tried even swimming or Aqua therapy and all, but I can’t go down without my AFO like if I have to get into water, then I have to remove the AFO. I’m scared that I’ll slip and fall.” (P1)*

#### Reinforcement

On what might positively or negatively reinforce PA, the greatest determinant was the effects gained with PA. Stroke survivors reported that the benefits of PA (i.e., opportunities to improve and to be better physically, mentally and emotionally) were what kept them going. Some reported a sense of achievement at seeing themselves improve (e.g., being able to walk independently with no aid), while others shared that the consequences of physical inactivity (e.g., weight gain, weakness, falls, diabetes) were what prevented them from stopping their PA. Opportunities to connect with others in a group were also reported by stroke survivors to be positive reinforcement to engage in more PA. Vouchers and financial incentives received mixed responses. While some stroke survivors welcomed financial incentives (provided they could put on the PA tracking devices with ease or could walk up to the recommended number of steps), some were ambivalent or did not find the financial incentives appealing, particularly when compared to the opportunities to improve and meet others for social connections.

*“I think it’s more intrinsic. If I’m active I’m able bodied, I can walk better, I think that’s the motivation for me. Cannot walk then I’m stuck in the house, that kind of thing, that’s very bad. That is my motivating point, to push myself such that I will walk for tomorrow.” (P15)*

*‘But then it [voucher/financial incentive] doesn’t appeal to me. I don’t know why, but it appealed to my sister there. I don’t know because like her age 60 over years old maybe she don’t work so she got more time doing all these collect points and walk.” (P4)*

#### Behavioural regulation

Most stroke survivors shared that they monitored or kept track of how much they engaged in PA using a mix of gadgets (e.g., Google Fit app on mobile phone, Garmin tracker on watch) and simple measures (e.g., duration and reps of an activity). However, in terms of how hard they worked during PA, most do not monitor this via objective measures but relied on subjective “feel” (i.e., stopping when they felt tired/pain or did not feel well). These findings were interesting to note and corroborated with earlier findings (in the *Knowledge* domain) regarding the lack of awareness of the recommended intensity of PA (i.e., how hard one should be working).

*“I have Google Fit app on my phone, on Steps app which counts my number of steps and how much I have walked.” (P1)*

*"I just go with the day’s flow so if I that day don’t feel too good or feel very tired. I don’t force myself to do it…” (P9)*

#### Beliefs about capabilities

Most stroke survivors reported having reduced confidence in engaging in more PA in terms of adapting exercises and using equipment. These beliefs were often associated with the presence of complications or other health challenges, the need for physical assistance during activities, and environmental barriers perceived to be out of their control. For the small number who expressed confidence in engaging in more PA, they did not require physical assistance to walk and were able to engage in PA on their own.

*“Yeah, vigorous exercise. I don’t think I can really ever do it, the only thing I can do is walk, walk and walk. There’s nothing much I can do.” (P12)*

*“I am not confident of using the treadmill. Prior to my stroke, I used to run…But now, I’m a little underconfident…Yes. I wish I could run again.” (P1)*

### Not relevant TDF domains

**Fig 2:**
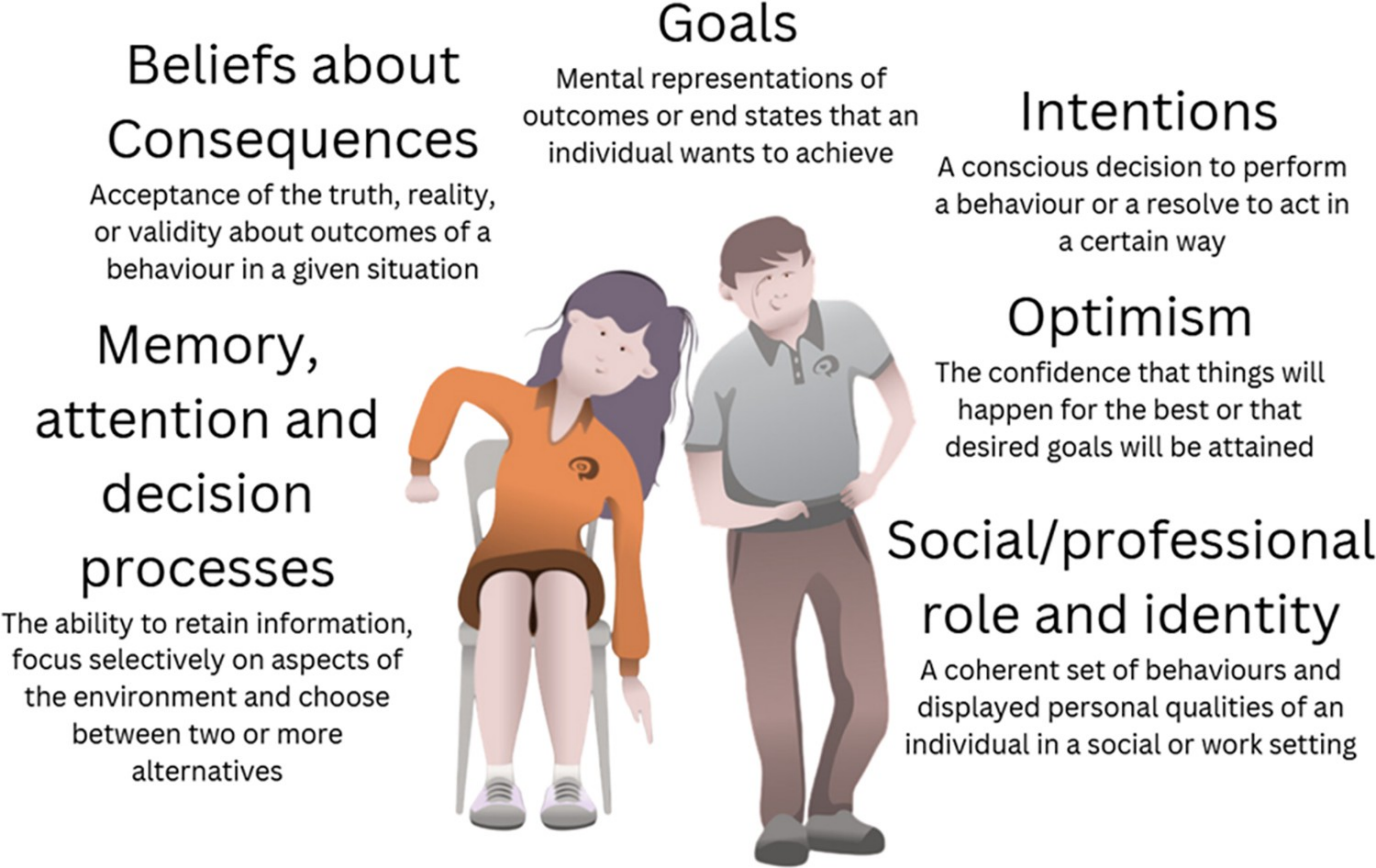


Six TDF domains were identified as not relevant (Fig 2). These domains were unlikely to influence behaviour and were considered of moderate and low importance (Table 2). These domains were beliefs about consequences, goals, intentions, optimism, social/professional role and identity, and memory, attention and decision processes (Table 4). Although domains such as goals, intentions and optimism had conflicting belief statements, they were considered of moderate importance due to low frequency and/or weak strength of belief statements. Conversely, beliefs about consequences had no conflicting belief statements but was high in frequency and strong in strength. The remaining domains of social/professional role and identity, and memory, attention and decision processes had no conflicting belief statements, and were weak in strength, and low in frequency and weak in strength respectively (Table 2).

**Table 4:** Not relevant belief statements and sample quotes tagged to the TDF domains.

TDF domains	Belief statements	Sample quotes (Participant number)	Frequency (out of 19)
**Beliefs about consequences**	If I become less physically active, I will incur the consequences.	“If I’m inactive then probably my spasticity will increase. Stiffness will increase and I’ll turn into a pumpkin. . .No, I like to meet people as I told you. So even my social interaction skills will come down […]” (P1) “If you are not active enough, again, you will find yourself getting lazier […] Then your mind, all this will start to shut down. What I mean is if you are not active at all, even once you are not active enough, I date you out I call you out you won’t go out. But if you are active about, you will okay let’s go. So this is the worst. So if your mind is not working, your activity all down. Correct. If your mind is working, somehow or whatsoever, you will still have something in your path.” (P7) “Like for example, the brain becomes slower and then there will be heart problem. And then hypertension which already I have. Also we’re trying to move to get it going and most important is I realise that by not moving it can have DVT which I’m having. And maybe another stroke.” (P16)	17
	If I become more physically active, I will reap the benefits.	“I believe that it will strengthen up my muscles, the strength. I think that’s why when I’m discharge from the rehab, the [name of organisation], I believe that most of the thing I need is to firstly to maintain. Secondly, if I can increase my strength, that’s all. And the rest, even the machine in the rehab centre whatever, is just assistant. I think back to a social society, all this normal daily life will help. Yeah I need to increase function as well as the standard of living better rather because those machine is the same thing, went to the cycling, is the same thing, it’s just to build up your strength and muscle.” (P6) “Swimming before I work, I really do to swimming because it helping me to build up my muscle.” (P19)	10
**Social/Professional role and identity**	I am personally responsible for my own health.	“No, not somebody’s. It’s our own [responsibility].” (P2) “I think it’s my responsibility. Nobody is responsible for our improvement if we don’t want to take, take into responsibility at the end of the day is we ourselves suffer.” (P9) “Of course, it’s your own life. You must be responsible for your own life, not somebody else, right?” (P19)	15
	PA reduces the stigma of being a stroke survivor.	“I feel more confident that I’m active. And probably that stigma of being a stroke survivor will lessen, reduce a lot.” (P1)	1
**Goals**	I have goals related to PA that I would like to achieve.	“I want to do a little more like I told you, aqua therapy and all I want to try […]” (P1) “Yeah, for sure. I mean I want to be walking better.. Of course, running it will be a optimal goal, but I want to be realistic also. So my partner she loves to go hiking. So the thing is, hiking is a challenge for me because it’s not even grounds. Up slope uneven grounds, up slope, down slope slope, so yeah. I’ve been to some of the nature reserves is actually not easy, but if since as long as there’s a real thing. railing for safety. So you can hold on to it. I think it’s okay.” (P3) “Cycling now at the moment I can’t do it. Trying to do it. My next goal is to cycle, to do a rock climbing…I’m still trying to get back to cycling. And rock climbing is because another of my handicapped friend which is an amputee. If he can do it, so I say you can do it I can do it also, so I’m going to try.” (P5)	10
	I have a clear plan on how often I will engage in more PA	“Yes, I’m looking into maybe another month or what I may have to go to walk outside this area, or maybe even walk to the lift lobby just with my quad stick. I think walking part should not be a problem. Or maybe initially I need someone to just bring a wheelchair with me in case I need to sit or rest at any time.” (P6) “Each time will be about an hour. And all the while, what I’ve been doing is about—need to strengthen my legs and I always strengthen my hands itself, by doing some of the things that I’ve been taught, where I were doing the therapies itself. And also […]when I’m back at home right, every day I will try to do whatever stretching and strengthening exercise that I have been taught to do itself. And also I will—actually when my mum need people to help her do some groceries, I mean, it’s not very heavy, it’s just like maybe like buying some stuff right? […] actually I will do my own analysing before I start my journey itself. […] And also, I also […] let’s say some of the journey right, instead of just walking right, I will actually take an initiative, climb the staircase. Ah journey, let’s say maybe my house, my house right, I stay at twelfth level, so I will try to walk down.” (P11)	4
	I have no goals related to PA that I would like to achieve.	“Try to do more. No, at the moment I didn’t set any of these kind of goal for myself. Yeah, no specific goal.” (P9)	1
**Intentions**	I intend to be stay/be more active in the near future.	“So I’m going to find ways and where is it and where is the right time and right place to do the thing. . .As I said, my next target is I wanted to ride on a bike and rock climbing. So these are things that I want to do. Need to plan who will be going with you. The equipment that you need to get. Just like if you want to ride on a bike, what type of bike that is suitable for us? Which bike should I buy? What are the things that I should do first before I grab on to actual bike, all these you need to plan. Because before you want to do, you gotta have the things to do it.” (P5) “I have been asking around how do I sign up for swimming lesson but nobody seems to know about it. They say that now with Covid everything is stopped. Because I used to be a swimmer and I love to swim so I’m hoping to see whether I can use back some function.” (P16)	6
	I do not intend to stay/be more active in the near future.	“[…] occasionally my staff ask me to go out. I also feel lazy to go. . .Because I habit. (P4) “I know that it’s important that we have to get ourselves fit. But the thing is sometimes due to our commitment, I mean whether you are able or disable, your work commitment, the other one is laziness.” (P7) “It should be, but now I have became so lazy. I’m just sitting down most of the time. Yeah, So what I do is that instead of being active and we’ll just do some art and craft, that do not involve too much movement. That’s it, stay put. And also because sometimes when you move, your body doesn’t listen to you and then you will find that the leg is not moving with you.” (P16)	4
**Optimism**	I do not feel optimistic about solving the problems limiting my PA.	“I think the chances [of being physically active] is probably less than 20%, unless I find some kind of alternative solution.” (P3) “Not that I can think of now because otherwise I will have done it. I have tried a few times with the stroke association. They also have virtual exercises, I have tried but just don’t motivate me.” (P4)	5
	I feel optimistic about solving the problems limiting my PA.	“It’s actually depend on how we think about it la, because some people—if let’s say I think that okay, I can’t be using this machine, all these all that right, they’re actually limited—I mean my ability to use it, but at the same time, if let’s say we think, think of other alternatives right? Actually, even though I don’t go to gym, I can do other thing, I mean at home itself. For example, for my leg right, I can see that squat, squatting up and down, even though I found like for me, I can do the squatting up and down itself. Also, I can be doing like some weight or maybe some other thing right, to—I mean to replace what the gym equipment is la, then I [inaudible] myself.” (P11) “I should say I would try, this is only I qualify to myself, I would try, but I cannot say that I will or I always want, sometimes I also may have my own limitation so I say I will try. OK. The thing about recovery, the expectation is always a straight line. But often the thing comes, goes and maybe turns around, come back again, so it is not like that. So people often think that 2 months down the road, you should be able to work, like my boss told me. . . hello, I’m having a stroke, not a fractured arm or what. Yeah, so the recovery is very dynamic. Correct, so you must allow for this back turn, forward turn, side turn whatever turn, all this disruption you come in before you can go ahead again. Yeah, because some days, you’re why am I like that, or why. . . you will begin to groan and moan. . . but can, I will be better tomorrow. Then you try to move on again.” (P15)	5
**Memory, attention and decision processes**	I seldom forget about staying/becoming more physically active.	“No, life goes on. Before my stroke, I’m very active. I’m normal. To me, I’m very normal. […] I don’t set the time or how many times must I do and all that. To me it’s like getting back to my daily life. Just like I fold clothes, I iron my clothes, you don’t say it’s how many amount of clothes that you have you need to iron. You need to finish, then you need to finish. So there’s no such thing as timing by one hour you must iron all these clothes, by one hour you must fold all these clothes. It’s like a normal life that you’re doing it.” (P5) “But generally because you go through that motion, Hmm take that moments of needs so it’s sort like it’s around that there about. In a way, routine […]” (P18)	4

#### Optimism

For a few stroke survivors, they were optimistic in finding solutions to their problems, often in part due to past experiences and strategies in adapting their activities, acceptance of the stroke recovery process, and having options and other networks to tap on (e.g., church groups). For others, they were less optimistic as the problems centred around their post-stroke complications (e.g., spasticity and not getting the right advice) and the COVID-19 pandemic, problems that were deemed to be beyond their control.

*“I think the chances [of being physically active] is probably less than 20%, unless I find some kind of alternative solution.” (P3)*

#### Intentions

While a few stroke survivors shared their intentions to engage in a new PA (with some clearly stating their goals and future plans), a few also reported feeling lazy and having reduced motivation to engage in more PA in light of work commitments and desire for more company during PA.

*“So I’m going to find ways and where is it and where is the right time and right place to do the thing…As I said, my next target is I wanted to ride on a bike and rock climbing. So these are things that I want to do. Need to plan who will be going with you. The equipment that you need to get. Just like if you want to ride on a bike, what type of bike that is suitable for us? Which bike should I buy? What are the things that I should do first before I grab on to actual bike, all these you need to plan. Because before you want to do, you gotta have the things to do it.” (P5)*

#### Goals

Most stroke survivors had goals which they wanted to achieve, be it trying new things (e.g., aqua therapy, running, cycling) or returning to their pre-morbid level of function (e.g., hiking, travelling). Few stroke survivors had clear action plans related to their PA goals. Only one individual had reported having no specific goals to work towards.

*“Yeah, for sure. I mean I want to be working walking better.. Of course, running it will be a optimal goal, but I want to be realistic also. So my partner she loves to go hiking. So the thing is, hiking is a challenge for me because it’s not even grounds. Up slope uneven grounds, up slope, down slope slope, so yeah. I’ve been to some of the nature reserves is actually not easy, but if since as long as there’s a real thing. railing for safety. So you can hold on to it. I think it’s okay.” (P3)*

#### Beliefs about consequences

All stroke survivors had accurate beliefs about the benefits of physical activity and consequences of physical inactivity. These included the effects of PA such as reducing the risk of stroke, reducing risk factors for stroke (e.g., blood pressure, cholesterol, blood sugar levels), managing complications (e.g., spasticity), reducing weight gain, reducing low mood, improving mental stimulation, preventing muscle atrophy and improving mobility.

*“Like for example, the brain becomes slower and then there will be heart problem. And then hypertension which already I have. Also we’re trying to move to get it going and most important is I realise that by not moving it can have DVT which I’m having. And maybe another stroke.” (P16)*

#### Social/professional role and identity

Stroke survivors reported that they were personally responsible for their own health, and saw being physically active as something they should be accountable for. They did not expect others to assume responsibility for them. One stroke survivor shared that being physically active helped to reduce the stigma of being a stroke survivor as she learnt to do more things on her own.

*“I think it’s my responsibility. Nobody is responsible for our improvement if we don’t want to take, take into responsibility at the end of the day is we ourselves suffer.” (P9)*

#### Memory, attention and decision processes

Few stroke survivors commented that they seldom forgot to engage in PA as these activities were now part of their routine everyday lives.

*“But generally because you go through that motion, Hmm take that moments of needs so it’s sort like it’s around that there about. In a way, routine […]” (P18)*

## Discussion

Our study is the first to report determinants to PA amongst stroke survivors with physical disabilities. Using a theoretical model of behaviour, we have identified eight relevant domains that are likely to improve PA levels after stroke if targeted in future interventions. These domains include environmental context and resources, knowledge, social influences, emotion, reinforcement, behavioural regulation, skills and beliefs about capabilities.

Similar to other studies in high income countries [7, 8, 12], the broad domains of environmental context and resources, social influences and beliefs about capabilities were highlighted in our cohort as key factors influencing PA after stroke. There was congruence between our data and that of other studies, as stroke survivors often reported having stroke-related impairments which affected their confidence in engaging in PA [8, 12], and the importance of a supportive network of friends and family to provide the psychological and social support to engage in PA [7, 8, 12]. Although the domains were broadly similar to other studies, there were two notable differences within the environmental context and resources domain.

First, the lack of transport and high cost of exercise programs were often featured under environmental factors influencing PA in the United States [9, 31] and United Kingdom [12]. In our study, stroke survivors reported greater concerns with access and use of public spaces for PA. In Singapore, lack of transport and high cost of exercise programs were likely less of an issue due to the widespread availability of public spaces to exercise. For example, public gyms are situated in most neighbourhoods [32], outdoor fitness zones are built near public housing flats [33] and the park connector network linking major parks and nature areas across Singapore offers a variety of routes and trails for walking and cycling [34]. The national initiative of according all Singapore citizens and permanent residents $100 credit to access public gyms and swimming pools [10] had also been useful, though it was apparent that stroke survivors used the $100 credit but were not able to access and use the facilities and equipment optimally (e.g., lack of ramp access in swimming pools limited easy access in and out of the swimming pools). Despite the rise of inclusive gyms and swimming pools in Singapore [35, 36], it appears that further solutions are needed for successful uptake. Some possible solutions offered by stroke survivors during the subsequent co-design workshops included raising awareness of inclusive sport facilities, running group exercises classes, and providing physical assistance and adaptive equipment (e.g., arm and leg straps) during the classes. These solutions are likely to have implications on intervention design for other people with physical disabilities.

Second, the impact of COVID-19 on stroke survivors’ PA levels was evident in our study as it was conducted during the pandemic while most studies looking at factors influencing PA were conducted before the pandemic [7, 8, 12]. Other than the COVID-19 restrictions which led to the cancellation of face-to-face exercise classes and social outings, and difficulty in securing gym bookings, stroke survivors found it hard adjusting to virtual activities and were more likely to stay at home in order to reduce the risk of infection. These factors highlight the need to consider country-specific factors in the current climate in order to develop targeted solutions for stroke survivors with physical disabilities to engage in PA after stroke.

Existing strategies and national initiatives targeted at improving PA in the general population were not accessible to stroke survivors with physical disabilities. In Singapore, there is a successful public health campaign termed the National Steps Challenge which uses trackers, rewards and mass media campaigns to engage the public in more PA [37]. To date, the campaign has registered 696 907 participants, engaged 60% of them in active participation for a median of 74 (IQR, 14–149) days, and increased mean daily step count by about 1579 (95% CI, 1564–1594) steps during the main challenge phase and 934 (95% CI, 916–952) steps during the maintenance phase. While some stroke survivors in our study could recall the importance of achieving 10,000 steps per day for health benefits (a possible outcome achieved via the public health campaign), some felt that the goal was unattainable and therefore did not partake in the campaign. When asked about incentives or reinforcements to stay active, the greatest determinant was the physical, mental and emotional benefits gained with PA, while vouchers and financial incentives received mixed responses. A previous nationwide survey in Singapore had identified the top three barriers to PA to be lack of time (65.3%), fatigue (64.7%) and pollution (56.1%) (n = 2867 participants sampled from a national administrative database of all residents in Singapore) [38]. These barriers differed greatly from the barriers to PA highlighted by stroke survivors in our study. Hence, it is likely that we need new strategies beyond the existing strategies and national initiatives to target stroke-specific barriers and improve PA after stroke in Singapore.

Using the recommended prioritisation criteria [19], we had identified eight TDF domains to be relevant (i.e., domains most likely to change behaviour if targeted in future interventions). To ensure we did not miss out any TDF domains that might have a significant influence on changing behaviour, we further rated the importance of each TDF domain as high, moderate or low importance depending on the number of criteria met [27], and discussed each domain and its potential influence on behaviour based on existing literature and data from our study. Of the remaining TDF domains of moderate and low importance (Table 2), we highlighted the intentions domain to be of potential influence due to the proven efficacy of implementation intention interventions in improving PA [39–41], presence of conflicting belief statements and the strength of belief statements from our data. According to the Theory of Planned Behaviour, intention refers to an individual’s willingness to act and predicts behaviour [42]. Although there remains an intention-behaviour gap (i.e., not all individuals with strong intention initiate the behaviour), implementation of intention interventions including action planning and coping planning are often incorporated to improve PA [39]. We are uncertain why the intentions domain was not relevant in our study, but the lack of motivation is not consistently reported in other studies on barriers to PA in the stroke population [8]. It is possible that in light of other problems such as stroke-related impairments, environmental barriers, low self-efficacy resulting in low engagement in PA, the lack of motivation becomes a smaller determinant to PA behaviour.

### Strengths and limitations

The strengths of our study include the use of a comprehensive theoretical model of behaviour [11, 19, 24], and the inclusion of stroke survivors with physical disabilities. Current studies often include stroke survivors with no physical disabilities [7, 8, 12], and most have not used behaviour change theories to underpin their results [7, 8]. Despite the strengths, our study had several limitations. First, our study focused predominantly on the viewpoints of stroke survivors and did not capture the viewpoints of caregivers. Considering the pivotal role that caregivers play in the recovery journey and their unique experiences of healthcare services [43], it is worthwhile to explore determinants to PA from the caregiver’s viewpoint in order to target barriers which caregivers might foresee when supporting stroke survivors in their PA journey. Second, barriers and facilitators reported by stroke survivors were perceived, and might not necessarily reflect the actual barriers and facilitators to PA experienced in real life. Third, all interviews were conducted in English. Although English remains the dominant language spoken at home in Singapore [44], at least half of Singaporeans spoke other languages including Mandarin, Chinese dialects, Malay, Tamil and others. Future studies should consider interviewing caregivers and including other methods of data collection such as observations in stroke survivors’ homes to provide a richer picture of stroke survivors’ PA levels and further contextualise interview data [45]. Resources should also be allocated to assist with the conduct, analyses and translation of interview data in different languages to reflect viewpoints of all stroke survivors in Singapore.

## Conclusions

In stroke survivors with physical disabilities, environmental context and resources had a significant influence on PA participation, and this often had a spill over effect into other domains. While some of our findings were similar to findings in other high-income countries, some factors were unique to the PA ecosystem in Singapore. Our results inform the development of a complex behaviour change intervention targeted at improving PA in stroke survivors with physical disabilities in Singapore.

## Supporting information

S1 File Interview guide. (DOCX)

S2 File Consolidated criteria for reporting qualitative studies (COREQ): 32-item checklist. (DOCX)

## Abbreviations

PA: Physical Activity
TDF: Theoretical Domains Framework
BCW: Behaviour Change Wheel
SNSA: Singapore National Stroke Association
