# Supplementary material for: What influences stroke survivors with physical disabilities to be physically active? A qualitative study informed by the Theoretical Domains Framework
Source: PLoS One. 2024 Mar 28;19(3):e0292442. doi: 10.1371/journal.pone.0292442 (PMC10977677; doi:10.1371/journal.pone.0292442)
Supplement: S1 File — (DOCX) [file pone.0292442.s001.docx]

**Supplementary materials 1 - Interview Guide**

Introduction: Hi, my name is [insert name]. I am a [insert role/profession] working on this project. Thank you for taking time out to participate in this study. The aim of this interview is to help us understand what helps, or stops you from being more active after your stroke. Being active can include doing exercises, or your daily activities (e.g., housework), whatever it is that gets you moving and makes you huff and puff. There is no right or wrong answers, as we are trying to understand how stroke survivors approach this issue, so please answer as honestly as you can.

Opening questions (broad):

Would you consider yourself as a physically active person?

What do you usually do to keep active?

Interview questions:

1. Do you know about the importance of being active?

*[Prompt(s): Do you think people after stroke should move just as much as healthy adults?*

*How many minutes of moderate-intensity physical activity do you think is recommended after stroke? (By moderate intensity, we mean activities/exercises that make you huff and puff.)*

*Are you aware of any physical activity guidelines for stroke survivors?]*

2. Do you know how to be active/more active?

*[Prompt(s): How confident are you in being more active (by that, I mean exercising/moving more/engaging in exercises/movements that make you huff and puff)?*

*What makes you confident? How confident are you in being more active?]*

3. Is being active something you usually do?

*[Prompt(s): Do you always remember to be more active?*

*When was the last time you recall having a conversation with someone about being more active/exercising more/moving more?]*

4. How do you monitor how much and how hard you have exercised/moved?

5. To what extent do your friends and family help or stop you from being active/more active?

*[Prompt(s): Do you feel you move/exercise more when other people are around than you do when you are alone? Or you feel it is the same?]*

6. When you wanted to be active/more active, how often did you feel you had the space, or equipment to do so?

*[Prompt(s): When you needed to move/exercise, how often did you feel you could do it at home, or go to a gym/outdoors fitness zone? (Every time - Most times - Sometimes – Rarely - Never)*

*When you needed to move/exercise, how often did you feel you have appropriate equipment to exercise? (Every time - Most times - Sometimes – Rarely - Never)]*

7. Do you see being active/more active as something that you should be personally responsible for?

*[Prompt: Do you think of yourself as someone who is a committed exercise person, casual exercise person, or not bothered either way about regular exercise?]*

8. How difficult or easy is it for you to do be active/more active?

*[Prompt(s): Do you think it is hard, or easy to be more active? What makes you say so?]*

9. How confident are you that the problem of being inactive/not moving will be solved?

*[Prompt: Do you feel optimistic/positive about being more active?]*

10. What do you think will happen if you do not become active/more active?

*[Prompt(s): Do you think about being more active?*

*What impact do you think being more active will have on your health?]*

11. Have you made a decision to be active/more active?

*[Prompt(s): Is being more active something that you generally intend to do?*

*On a scale of 0-5 (with 0 being no effort, and 5 being most effort you have ever exerted, how effortful do you feel it is to be more active?]*

12. How much do you want to be active?

*[Prompt: To what extent is being more active a priority for you?]*

13. Are there incentives to be active?

*[Prompt(s): Would you say that generally you are in the habit of being more active? If no, what would be helpful in developing a routine/habit to be more active?]*

14. Would being active make you feel good or bad?

*[Prompt: How does being more active make you feel?]*

[Participants are asked if they have further comments to add to any of their questions. If there are none, interviewer ends the interview.] Thank you so much for your time and responses.
