# Supplementary material for: What influences stroke survivors with physical disabilities to be physically active? A qualitative study informed by the Theoretical Domains Framework
Source: PLoS One. 2024 Mar 28;19(3):e0292442. doi: 10.1371/journal.pone.0292442 (PMC10977677; doi:10.1371/journal.pone.0292442)
Supplement: S2 File — (DOCX) [file pone.0292442.s002.docx]

**Supplementary materials 2 - Consolidated criteria for reporting qualitative studies (COREQ): 32-item checklist**

| **No.** | **Item** | **Guide questions/description** | **Page number and lines** |
| --- | --- | --- | --- |
| **Domain 1: Research team and reﬂexivity** | | | |
| Personal Characteristics | | | |
| 1. | Interviewer/facilitator | Which author/s conducted the interview or focus group? | p.8, lines 181-183 |
| 2. | Credentials | What were the researcher’s credentials? *E.g. PhD, MD* | p.8, lines 183-186 |
| 3. | Occupation | What was their occupation at the time of the study? | p.8, lines 183-186 |
| 4. | Gender | Was the researcher male or female? | p.8, lines 184-185 |
| 5. | Experience and training | What experience or training did the researcher have? | p.8-9, lines 181-200 |
| Relationship with participants | | | |
| 6. | Relationship established | Was a relationship established prior to study commencement? | p.8, lines 186-193 |
| 7. | Participant knowledge of the interviewer | What did the participants know about the researcher? *E.g. personal goals, reasons for doing the research* | p.8, lines 186-193 |
| 8. | Interviewer characteristics | What characteristics were reported about the interviewer/facilitator? *E.g. Bias, assumptions,*  *reasons and interests in the research topic* | p.8, lines 181-193 |
| **Domain 2: study design** | | | |
| Theoretical framework | | | |
| 9. | Methodological orientation and  Theory | What methodological orientation was stated to underpin the study? *E.g. grounded theory,*  *discourse analysis, ethnography, phenomenology, content analysis* | p.9, lines 203-228 |
| Participant selection | | | |
| 10. | Sampling | How were participants selected? *E.g. purposive, convenience, consecutive, snowball* | p.6, lines 128-137 |
| 11. | Method of approach | How were participants approached? *E.g. face-to-face, telephone, mail, email* | p.6, lines 128-137 |
| 12. | Sample size | How many participants were in the study? | p.9, lines 210-212 |
| 13. | Non-participation | How many people refused to participate or dropped out? Reasons? | p.9, lines 212-214 |
| Setting | | | |
| 14. | Setting of data collection | Where was the data collected? *E.g. home, clinic, workplace* | p.6, lines 139-145 |
| 15. | Presence of non-participants | Was anyone else present besides the participants and researchers? | p.6, lines 141-144  p.7, lines 164-166 |
| 16. | Description of sample | What are the important characteristics of the sample? *E.g. demographic data, date* | p.9, lines 210-220;  Table 1 |
| Data collection | | | |
| 17. | Interview guide | Were questions, prompts, guides provided by the authors? Was it pilot tested? | Supp file 1 |
| 18. | Repeat interviews | Were repeat interviews carried out? If yes, how many? | NA |
| 19. | Audio/visual recording | Did the research use audio or visual recording to collect the data? | p.7, lines 170-173 |
| 20. | Field notes | Were ﬁeld notes made during and/or after the interview or focus group? | p.7, lines 171-172 |
| 21. | Duration | What was the duration of the interviews or focus group? | p.10, lines 242-243 |
| 22. | Data saturation | Was data saturation discussed? | p.6, lines 144-147 p.10, lines 244-247; |
| 23. | Transcripts returned | Were transcripts returned to participants for comment and/or correction? | p.10, lines 243-244 |
| **Domain 3: analysis and ﬁndings** | | | |
| Data analysis | | | |
| 24. | Number of data coders | How many data coders coded the data? | p.9, lines 203-216 |
| 25. | Description of the coding tree | Did authors provide a description of the coding tree? | p.9, lines 203-222 |
| 26. | Derivation of themes | Were themes identiﬁed in advance or derived from the data? | p.9, lines 203-216 |
| 27. | Software | What software, if applicable, was used to manage the data? | p.10, lines 228-229 |
| 28. | Participant checking | Did participants provide feedback on the ﬁndings? | p.10, lines 243-244 |
| Reporting | | | |
| 29. | Quotations presented | Were participant quotations presented to illustrate the themes / ﬁndings? Was each  quotation identiﬁed? *E.g. participant number* | p.12-21, and Tables 3 and 4 |
| 30. | Data and ﬁndings consistent | Was there consistency between the data presented and the ﬁndings? | p.11-21, and  Tables 2, 3 and 4 |
| 31. | Clarity of major themes | Were major themes clearly presented in the ﬁndings? | Tables 2 and3, and Figure 1 |
| 32. | Clarity of minor themes | Is there a description of diverse cases or discussion of minor themes? | Tables 2and 4, and Figure 2 |
